# Le nævus congénital géant

**DOI:** 10.11604/pamj.2014.18.48.4468

**Published:** 2014-05-14

**Authors:** Majdouline Boujoual, Fatima Jabouirik

**Affiliations:** 1 Gynécologie Obstétrique, CHU Ibn Sina, Rabat, Maroc; 2 Pédiatrie, CHU Ibn Sina, Rabat, Maroc

**Keywords:** Nævus congénital, lésion cutanée, mélanome, congenital nevus, skin lesion, melanoma

## Image en medicine

Le Nævus congénital géant est une lésion cutanée pigmentée dont la taille ≥ la paume de la main (1% de la surface corporelle). Son incidence est de 1/20.000 naissances. Son diagnostic est clinique: Il s'agit d'un placard cutané circonscrit de teinte brune, souvent recouvert de pilosité dense ou de nodules de prolifération et/ou accompagné de nævi satellites. Il peut se compliquer d'une mélanocytoseleptoméningée, ou s'associer à d'autres tumeurs neurectodermiques de sévérité variable. Son caractère géant augmente le risque de dégénérescence en mélanome, alors que son caractère inesthétique et sa surface irrégulière gênent souvent la surveillance dermatologique, justifiant alors son exérèse préventive précoce. Ailleurs, la Dermabrasion, le curetage et le laser peuvent être des alternatives efficaces. Nous illustrons le cas d'un nouveau-né à J1 de vie, issu d'une grossesse menée à terme, présentant une plaque pigmentée faisant environ 30/20 cm, occupant le flanc droit et étendue jusqu’à l'ombilic, d'aspect hétérogène parsemé par endroits de quelques espaces sains, de texture tantôt lisse et tantôt épaisse et de contours nets. Le reste de l'examen somatique était sans particularités. Il a été adressé en Chirurgie Plastique pourune prise en charge thérapeutique.

**Figure 1:**
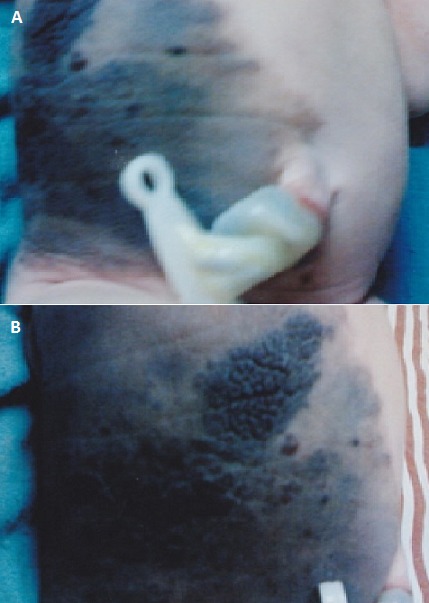
A) nævus congénital géant du flanc droit; B) aspect géant et hétérogène du nævus.

